# List of Original Papers in the British Medical Journals for the Quarter Ending the 20th March, 1843

**Published:** 1843-04

**Authors:** 


### ORIGINAL PAPERS IN THE BRITISH MEDICAL JOURNALS.

For the Quarter ending 20th March 1843.

---

### *Anatomy, Physiology, and Morbid Anatomy.*

Mr. J. Allison—Case of Acephalous Fetus . . *Prov. Jour.* Dec. 17.
Dr. S. Fife—Diagnosis of Cholera . . . . *Ibid.* Dec. 31.
Dr. A. S. Malcolm—On the Formation of Carbonic Acid in Respiration in Typhus *Edin. Monthly Jour.* Jan. 1.
Mr. R. Nasmyth—On the Physiology and Pathology of the Teeth *Ibid.* Jan. 1.
Dr. Haygarth—On the Function of the Spleen . . . *Med. Gaz.* Jan. 13.
Mr. Davis—Imperforate Pharynx, &c. . . . *Ibid.* Jan. 13.
Dr. Power—On the Fibres of the Optic Nerve . . *Dublin Jour.* Jan. 1.
Dr. Stratton—On the Morning and Evening Pulse . . *Edin. Jour.* Jan. 1.
Dr. Holland—On the State of the Blood in the Veins . *Ibid.* Jan. 1.
Dr. Reid—On the Blood-vessels of Mother and Fetus . *Ibid.* Jan. 1.
Dr. Pring—On the Evolution of Animal Electricity . *Medical Gazette,* Jan. 6.
Mr. Ellis—On the Posterior Divisions of the Spinal Nerves *Ibid.* Feb. 10.
Mr. Snow—On the Capillary Circulation . . *Ibid.* March 3.
Dr. Ayres—On the Physiology of Disease . . *Lancet,* March 11.
Mr. Labatt—On a Valve in the Vena Cava . . *Dublin Press,* Feb. 22.

### *Pathology, Practical Medicine, and Therapeutics.*

Dr. Hooper—Case of Pneumonia . . *Prov. Med. Jour.* Feb. 18.
Mr. Griffith—On Convulsions in Delirium Tremens *Ibid.* Feb. 25.
Mr. Miller—Case of Catalepsy in Mania . . *Ibid.* March 4.
Dr. Fife—On the Nervous Symptoms in Cholera . . *Ibid.* March 11.
Mr. Lee—On Indian Hemp . . . . *Ibid.* March 18.
Dr. Black—On the Topical Pathology of the Neuralgiæ . *Ibid.* March 11.
Sir H. March—On Strumous Peritonitis . . . *Dub. Journ.* March 1.
Dr. Aldridge—On Urinary Diseases . . . *Ibid.* March 1.
Dr. Reid—Case of Disease of the Spinal Cord . *Edin. Month. Jour.* March 1.
Dr. Reid—On Partial Hypertrophy of the Organ of Voluntary Motion. *Ibid.* March 1.
Dr. Graham—On Discoloration of the Skin by Nitrate of Silver *Dublin Press,* Feb. 1.
Dr. Clay—On the Diseases of Cotton Factories . . *Med. Times,* Feb. 4.
Dr. Willis—On the Diseases of the Heart and great Vessels. *Med. Gaz.* Feb. 3. et seq.
Dr. Page—On Electro-magnetism in Poisoning and Asphyxia . *Lancet,* Feb. 4.
Mr. Bolton—Case of Encysted Abscess of the Brain . . *Ibid.* Feb. 4.
Dr. O'Shaughnessy—On Indian Hemp . . *Prov. Journ.* Jan. 28.
Mr. Thompson—Case of Peritonitis, &c. . . . . *Lancet,* Jan. 28.
Dr. Hunter—On the Cold Water Cure . . . *Prov. Journ.* Feb. 4.
Mr. Ramsey—On the Pathology of Fever . . . *Med. Gaz.* Feb. 10.
Dr. Baly—On the Prevention of Scurvy in Prisons . . *Ibid.* Feb. 10.
Dr. Percy—Case of Malignant Tumours . . . *Ibid.* Feb. 10.
Dr. Fife—On Cholera . . . . . *Prov. Journ.* Jan. 21.
Dr. Hastings—On the Water Cure . . . . *Ibid.* Jan. 21.
Dr. Maddock—On the Coexistence of Vaccinia and Variola . *Lancet,* Feb. 11.
Dr. Dick—Case of Stercoraceous Vomiting . . *Med. Gaz.* March 10.
Dr. Willis—Ou Diseases of the Heart and Vessels . . *Ibid.* March 10.
Dr. Franz—Treatment of Hoarseness by Croton Oil . *Ibid.* March 3.
Dr. Maclachlan—On a peculiar Cardiac Sound . . *Ibid.* March 3.
Dr. Percy—Case of Albuminous Urine . . . *Ibid.* March 3.
Mr. Harrison—On Diffuse Carcinoma . . . *Ibid.* Feb. 24.
Mr. Thompson—On the supposed Influence of the Moon's Rays as a Cause of Disease *Ibid.* Feb. 24.
Mr. Ross—On Typhus Fever . . . . . *Lancet,* Feb. 25.
Dr. J. Waters—Efficacy of Tobacco Injections . . *Prov. Journ.* Dec. 17.
Mr. Gibson—On Diagnosis of Chest Diseases . . . *Lancet,* Dec. 24.
Mr. Solly—Case of Mollities Osseum . . . *Med. Times,* Dec. 24.
Dr. C. Bryce—Case of Lead-shot swallowed . . . *Lancet,* Dec. 31.
Dr. G. P. Smith—Case of Abscess of the Brain . . . *Ibid.* Dec. 31.

| | | |
|---|---|---|
| Dr. Allnatt—On Creosote in Leucorrhœa | *Lancet,* | Dec. 31. |
| Dr. Hocken—On Asthenic Amaurosis | *Ed. Month. Journ.* | Jan. 1. |
| Dr. Golding Bird—On Abdominal Neuralgia | *Ibid.* | Jan. 1. |
| Mr. Semple—Cases of Diseases of the Heart | *Lancet,* | Jan. 14. |
| Mr. Boden—Case of Perforation of the Stomach | *Ibid.* | Jan. 14. |
| Dr. Bow—On Blisters, &c. in Fever | *Ibid.* | Jan. 14. |
| Dr. Clay—On the Diseases of Cotton Factories | *Med. Times,* | Dec. 31, Jan. 14. |
| Dr. Ferguson—On the Propagation of Plague | *Edin. Journ.* | Jan. 1. |
| Dr. Boyd—Pathological Contributions | *Ibid.* | Jan. 1. |
| Dr. Hocken—On Hectic Fever | *Ibid.* | Jan. 1. |
| Dr. Thomson—Effects of Fatigue in India | *Ibid.* | Jan. 1. |
| Dr. Paterson—On Spectral Illusions | *Ibid.* | Jan. 1. |
| Sir D. Dickson—Case of Ascites | *Ibid.* | Jan. 1. |
| Dr. Craigie—Acute Farcy in Man | *Ibid.* | Jan. 1. |
| Dr. Smyth—On Impotence and Sterility | *Lancet,* | Jan. 7. |
| Dr. Stocker—Hydrocephalus after Scarlatina | *Prov. Journ.* | Jan. 14. |
| Dr. Lynch—On Delirium Ebriorum simulating Pleurisy | *Ibid.* | Feb. 11. |
| Dr. Ure—On Preventing the Phosphatic Diathesis | *Ibid.* | Feb. 11. |

*Surgery, including Diseases of the Eye and Ear.*

| | | |
|---|---|---|
| Dr. Lanyon—On Nitrate of Silver in Erythema | *Lancet,* | Jan. 21. |
| Mr. Stafford—On Mortification | *Med. Gaz.* | Feb. 3. |
| Dr. Dick—On the Pathology of Burns | *Ibid.* | Jan. 27. |
| Dr. Lawrie—On Aneurism | *Ibid.* | Jan. 27. |
| Mr. Bell—Fatal Hemorrhage from swallowing a Needle | *Ibid.* | Feb. 10. |
| Mr. Syme—Surgical Cases and Observations | *Ed. Month. Journ.* | Feb. 1. |
| Dr. Dunsmure—Cases of Tracheotomy | *Ibid.* | Feb. 1. |
| Dr. Paul—Compound Fracture of the Leg, &c. | *Ibid.* | Feb. 1. |
| Mr. Banner—Case of Fracture of the Skull | *Prov. Med. Journ.* | Jan. 21. |
| Mr. Wraith—Case of Cæsarean Section | *Ibid.* | Jan. 21. |
| Mr. Bryan—On a new Lithotomy Staff | *Lancet,* | Feb. 11. |
| Mr. Sankey—On outward Applications to Ulcers | *Med. Gaz.* | March 10. |
| Mr. Travers, jun.—Case of Extirpated Tumour | *Ibid.* | Feb. 24. |
| Dr. Reid—Treatment of Vesico-vaginal Fistula | *Lancet,* | Feb. 18. |
| Mr. Walker—On Capsular Cataract | *Prov. Med. Journ.* | Feb. 18. |
| Mr. Banner—On Fracture of the Long Bones | *Ibid.* | Feb. 28. |
| Dr. Houston—On Nitric Acid in Hemorrhoids | *Dub. Journ.* | March 1. |
| Mr. King—Rupture of the Jugular into an Abscess | *Ed. Month. Journ.* | March 1. |
| Dr. Hall—On Diseases of the Eye | *Med. Gaz.* | March 17. |
| Mr. Toogood—Wound of the Abdomen | *Prov. Journ.* | Dec. 17. |
| Dr. Laycock—Case of Ichthyosis | *Dub. Press,* | Dec. 21. |
| Mr. P. Lyon—Use of the Tourniquet in Venesection | *Lancet,* | Dec. 24. |
| Mr. J. Prankerd—Case of Femoral Hernia | *Prov. Journ.* | Dec. 31. |
| Mr. Hawkins—On Strangulated Hernia in Infants | *Med. Gaz.* | Dec. 30. |
| Dr. T. Howden—Case of Crural Hernia, with Perforation | *Lancet,* | Dec. 31. |
| Dr. F. W. Henderson—Case of Fracture in old age | *Med. Gaz.* | Jan. 13. |
| Mr. Paterson—On Prussic Acid in diseased Cornea | *Ibid.* | Jan. 13. |
| Mr. Erichsen—On the Pathology of Burns | *Ibid.* | Jan. 13. |
| Dr. Cusack—On Cleft Palate | *Dub. Journ.* | Jan. 1. |
| Dr. Bellingham—Aneurism of the Iliac Artery | *Ibid.* | Jan. 1. |
| Mr. Banner—Report of Fractures at Liverpool | *Ed. Journ.* | Jan. 1. |
| Dr. Adolphus—Cases in Surgery | *Ibid.* | Jan. 1. |
| Sir D. Dickson—Case of Aortal Aneurism | *Ibid.* | Jan. 1. |
| Mr. Elliott—Case of Calculus.—Cystectacy | *Ibid.* | Jan. 1. |
| Mr. Walne—Extirpation of the Ovary | *Med. Gaz.* | Dec. 23. |
| Dr. Douglas—Recovery from Asphyxia | *Ibid.* | Dec. 23. |
| Mr. Phillips—On Seminal Discharges | *Ibid.* | Dec. 23. |
| Mr. Solly—Case of Mollities Ossium | *Ibid.* | Jan. 6. |
| Dr. Thomson—On Blindness from Oil of Vitriol | *Ibid.* | Jan. 6. |
| Dr. Maclean—On Prussic Acid in Eye Diseases | *Lancet,* | Jan. 7. |
| Dr. Burgess—On Diseases of the Skin | *Prov. Journ.* | Jan. 14. |
| Mr. Jerrard—Case of Wound in the Abdomen | *Ibid.* | Feb. 11. |
| Mr. Phillips—On Seminal Discharges | *Med. Gaz.* | Jan. 20. |
| Mr. Lewis—Use of Iron in Gleet | *Lancet,* | Jan. 21. |

### *Midwifery, and Diseases of Women and Children.*

Mr. J. Waddington—Nursery Treatment of Infants . . *Lancet,* Dec. 29.
Mr. Tubbs—Effect of moral impressions on the Fetus . *Prov. Journ.* Dec. 31.
Dr. Granville—On the Removal of the Ovary . . . *Med. Gaz.* Jan. 13.
Dr. Camps—On Uterine Hemorrhage . . . *Ibid.* Jan. 13.
Dr. Macdonald—On Polypus of the Uterus . . . *Lancet,* Jan. 14.
Mr. Vale—On Twin Children born at different times . . *Ibid.* Jan. 14.
Dr. Mitchell—Contributions to Obstetric Medicine . . *Dub. Journ.* Jan. 1.
Mr. Pretty—External Pressure on Uterine Hemorrhage . *Med. Gaz.* Dec. 23.
Mr. Hunter—Table of Midwifery Cases . . . *Lancet,* Jan. 7.
Mr. Dorrington—Abdominal Apoplexy in Children . *Prov. Journ.* Jan. 14.
Dr. Macken—On Section of the Perineum . . . *Lancet,* Feb. 4.
Mr. Turner—On the Abdominal Line, as a sign of Delivery *Ed. Month. J.* Feb. 1.
Mr. Clewe—Case of Hydatids of the Uterus . . *Prov. Journ.* Jan. 21.
Dr. Holt—On the Ill Effects of Ergot . . . *Lancet,* Feb. 11.
Dr. Henderson—Strangulation of the child by the Os Uteri . *Ibid.* Feb. 11.
Mr. Girdwood—On the Theory of Menstruation . . *Ibid.* March 4.
Mr. Butcher—Case of Tapping of the Brain in Hydrocephalus *Dub. Journ.* March 1.
Mr. Renaud—Observation on the Placenta . . *Ed. M. J.* March 1.

### *Medical Jurisprudence, and Hygiene.*

Mr. Lafarque—Poisoning from Lucifer Matches . *Prov. Journ.* Dec. 24.
Mr. Tapson—Case of Poisoning by Oxalic Acid . *Med. Gaz.* Dec. 30.
Mr. Illingworth—Poisoning by Corrosive Sublimate . . *Ibid.* Jan. 13.
Mr. Garden—Temporary Poisoning from Hydrocyanic Acid *Edin. Journ.* Jan. 1.
Mr. Balfour—On the Effects of Irrigation on health in India. *Ed. Month. Jour.* Feb. 1.
Dr. Cormack—On Mechanical Inflation of the Lungs . . *Ibid.* March 1.

### *Materia Medica, Pharmacy, and Chemistry.*

Mr. J. B. Harrison—On the Inorganic Constituents of Organic Bodies. *M. Gaz.* Dec. 30.
Dr. Kemp—On the Composition of the Bile . . . *Med. Gaz.* March 3.

### *Medical Statistics, and Miscellaneous.*

Mr. Dodd—On the Medical Collegiate System . . *Med. Gaz.* Dec. 30.
Dr. Guy—King's College Hospital Report for 1842 . . *Ibid.* March 10.

